# State dependence of arousal from torpor in brown long-eared bats (*Plecotus auritus*)

**DOI:** 10.1007/s00360-022-01451-8

**Published:** 2022-08-16

**Authors:** Rune Sørås, Mari Aas Fjelldal, Claus Bech, Jeroen van der Kooij, Karoline H. Skåra, Katrine Eldegard, Clare Stawski

**Affiliations:** 1 Department of Biology, Norwegian University of Science and Technology, 7491 Trondheim, NO Norway; 2 Nature Education, Research and Consultancy van der Kooij, Rudsteinveien 67, 1480 Slattum, NO Norway; 3 Centre for Fertility and Health, Norwegian Institute of Public Health, Skøyen, P.O. Box 222, Oslo, 0213 NO Norway; 4 Faculty of Environmental Sciences and Natural Resource Management, Norwegian University of Life Sciences, Box 5003, 1433 Ås, NO Norway; 5 School of Science, Technology and Engineering, University of the Sunshine Coast, Maroochydore DC, QLD 4558 Australia

**Keywords:** Respirometry, Torpor, Vespertilionidae, Chiroptera, Temperature, Body mass

## Abstract

To cope with periods of low food availability and unsuitable environmental conditions (e.g., short photoperiod or challenging weather), many heterothermic mammals can readily go into torpor to save energy. However, torpor also entails several potential costs, and quantitative energetics can, therefore, be influenced by the individual state, such as available energy reserves. We studied the thermal energetics of brown long-eared bats (*Plecotus auritus*) in the northern part of its distributional range, including torpor entry, thermoregulatory ability during torpor and how they responded metabolically to an increasing ambient temperature (*T*_a_) during arousal from torpor. Torpor entry occurred later in bats with higher body mass (*M*_b_). During torpor, only 10 out of 21 bats increased oxygen consumption (*V̇*O_2_) to a greater extent above the mean torpor metabolic rates (TMR) when exposed to low *T*_a_*.* The slope of the torpid thermoregulatory curve was shallower than that of resting metabolic rate (RMR) during normothermic conditions, indicating a higher thermal insulation during torpor. During exposure to an increasing *T*_a_, all bats increased metabolic rate exponentially, but the bats with higher *M*_b_ aroused at a lower *T*_a_ than those with lower *M*_b_. In bats with low *M*_b_, arousal was postponed to an *T*_a_ above the lower critical temperature of the thermoneutral zone. Our results demonstrate that physiological traits, which are often considered fixed, can be more flexible than previously assumed and vary with individual state. Thus, future studies of thermal physiology should to a greater extent take individual state-dependent effects into account.

**Supplementary Information:**

The online version contains supplementary material available at 10.1007/s00360-022-01451-8.

## Introduction

Many endothermic animals have a remarkable capacity to alter their metabolic rate (MR) and heart rate, and thereby body temperature (Schmidt-Nielsen 1997; Currie et al. 2014). By lowering their energy consumption to the bare minimum for survival, some heterothermic endotherms can reduce their MR by up to ~ 1000-fold compared to their active metabolism (Willis et al. 2005) when entering the energy saving state of torpor (Geiser 2021). The large potential energy savings gained from employing short-term torpor or hibernation during inclement conditions have been found to enhance individual survival probability and even reduce the risk of species extinctions (Geiser and Turbill 2009; Liow et al. 2009). Torpor is primarily present and most pronounced in small mammals such as rodents (Buck and Barnes 2000; Zervanos et al. 2010; Pretzlaff et al. 2021), marsupials (Franco et al. 2012), and bats (Dunbar and Tomasi 2006; Jonasson and Willis 2012; McGuire et al. 2021), as well as some birds (Wolf et al. 2020; Geiser 2021). As an adaptation to various conditions, the use of torpor is prevalent during both summer and winter, and in both the Southern and Northern hemispheres (Stawski et al. 2014; Ruf and Geiser 2015; Boyles et al. 2016; Geiser 2021; McGuire et al. 2021; Reher and Dausmann 2021).

For small mammals with a large surface area to volume ratio, obtaining enough energy to maintain a stable body temperature (*T*_b_) is particularly challenging if they depend on a limited and seasonal food source (Buck and Barnes 2000), and employing torpor is a widely used strategy to cope with these challenges. At higher northern latitudes, the need for employing torpor on a day-to-day basis is further emphasized in nocturnal animals, as the length of summer night decreases with increasing latitude (Michaelsen et al. 2011). This means that nocturnal animals at high latitudes will need to build up sufficient energy reserves on a daily basis to compensate for the long days, and thereby long time between foraging bouts. Thus, the ability to enter torpor for shorter periods of time may be an essential strategy to both survive and reproduce during summers at high latitudes.

As torpor is not cost-free (Humphries et al. 2003; Boyles et al. 2020), there is a trade-off between the costs of maintaining torpor and the benefits of staying euthermic. Costs related to torpor include sleep deprivation (Humphries et al. 2003), memory loss (Millesi et al. 2001), and risk of predation (Estok et al. 2010; Haarsma and Kaal 2016), although the severity of the latter has been questioned (Turbill et al. 2011). In contrast, the benefits of maintaining a high *T*_b_ during daytime in summer include digestion of previously consumed food (Turbill et al. 2008) and allow for the development of fetus and lactation, which will be greatly delayed or reduced during torpor (Kurta et al. 1989; Dzal and Brigham 2013; Stawski et al. 2014).

It has recently been suggested that torpor is a more flexible trait than previously assumed (Reher et al. 2022). For example, torpor entry is often delayed (Matheson et al. 2010), and torpor duration decreased (Geiser and Broome 1993) in recently fed animals. Insectivorous bats (Chiroptera) at higher latitudes have a short reproductive season (Frafjord 2021), and rely on a food source which varies seasonally and occurs irregularly (Selås et al. 2013). Given their small body size, loss of heat is a major challenge when bats are faced with *T*_a_s below the thermoneutral zone (TNZ) (Bartels et al. 1998). Their limited potential for fat storage means that a substantial reduction of metabolic rate is the only possible option for non-migratory species when faced with longer periods of food shortage during winter at northern latitudes (Wermundsen and Siivonen 2010). Nevertheless, when and to which extent bats utilize torpor differs between species and environmental conditions (Stawski and Geiser 2010; Boyles et al. 2017), such as extreme heat (Reher and Dausmann 2021), unpredictable weather (Downs et al. 2012), or less suitable foraging conditions (Geiser et al. 2018).

As torpid bats may thermoregulate to some extent for short relocations within roost despite having a low *T*_b_ (Bartonička et al. 2017; Mayberry et al. 2017), it is reasonable to assume that there is an individual state component in studies investigating metabolic rate in bats that is often overlooked. Hence, as the cost of arousal increases with lower *T*_a_ (Wojciechowski et al. 2007), only bats with a higher energy reserve or better food availability can be expected to arouse at lower temperatures to counteract costs related to torpor (see Landes et al. 2020). When energy reserves are at a particularly low level, bats may not be able to arouse at low *T*_a_ as energetic reserves are insufficient to fuel arousal.

The brown long-eared bat (*Plecotus auritus*) is a medium sized (6–9 g) insectivorous Vespertilionid bat distributed widely across the Western Palearctic region (Wilson and Mittermeier 2019). Because the amount and availability of their prey is drastically reduced during the winter season, individuals must reduce their energy consumption to be able to hibernate through the winter. Towards the northern range of its distribution, *P. auritus* may also benefit from using torpor on a day-to-day basis throughout the year as night length, and thereby foraging opportunities, decrease with increasing latitude in summer (Michaelsen et al. 2011).

Some studies measuring metabolic rate have already been conducted within two populations of *P. auritus* in the western margin of its distribution range (Speakman et al. 1991; Webb et al. 1992; McLean and Speakman 2000; Becker et al. 2012, 2013). As *P. auritus* at high latitude are likely to enter short-term torpor on a day-to-day basis, understanding the cost and timing of arousal can improve our understanding of how bats survive in the northern hemisphere. Hence, in the present study, we experimentally studied this by inducing torpor in *P. auritus* and studied the physiology of torpor and the arousal from torpor. We predicted that individuals with more energy reserves should arouse earlier to reduce torpor-related costs, while those with low energy reserves would remain torpid for a longer period of time. We also predicted that *P. auritus* with larger energy reserves would utilize more energy to thermoregulate during torpor to reduce the costs and risks associated with deep torpor at low *T*_a_.

## Materials and methods

Bats exiting both potential and known roosts, or commuting along important flyways, were captured using mist-nets in Nittedal, Norway (60° 4′ 23″ N, 10° 52′ 20″ E) in June and July from 2019 to 2021. Upon capture, bats were immediately put into individual cloth bags, before measuring *M*_b_ to the nearest 0.1 g (Aweigh MB-50), forearm length to the nearest 0.1 mm (RS PRO 150 mm Digital Caliper 0.03 mm), while sex and reproductive state were determined. *M*_b_ was used as a proxy for energy reserves. Additionally, the wings of all captured bats were photographed using a standard DSLR camera with predetermined settings (1/160 s, *f*16, ISO 100), and an external flash providing back light to use the wing membrane for individual identification (see Amelon et al. 2017). Female bats that showed signs of reproduction (i.e., palpated abdomen or signs of lactation) were released after the individual morphometric measures. All males for which MR was measured were captured between June 1st and July 20th and did not show any signs of spermatogenesis.

After examination in the field, bats (*N* = 22) which were not reproductively active or not born the same year (i.e., closed epiphyseal gap) were brought back to an outdoor flight cage (2.5 m × 5 m × 2 m), which was equipped with bat boxes for roosting. Two of the four walls consisted of mesh netting, giving the cage an open air supply and a natural variation in light and environmental temperature. All bats were adult and consisted of 9 females and 13 males. On eight occasions, a single bat was brought back. Whereas, on some occasions, two (*N* = 5) or four (*N* = 1) bats were brought back at the same time. On one occasion, four bats were brought back in groups of two on two consecutive days. The following day, one bat was used for the experiment, while the other(s) were handfed *Tenebrio molitor* twice a day and given water ad libitum. Bats were always handfed before 21:00, so that they would be post-absorptive when the experiment started. When multiple bats were brought back the same day, females were always measured the following day, while males were measured on the 2nd day. Each bat was released at the capture site after sunset the same day the measurement had been performed. Thus, bats were held in captivity for 1 (*N* = 13), 2 (*N* = 5), 3 (*N* = 3) and 4 days (*N* = 1).

We measured metabolic rate indirectly as *V̇*O_2_ using open-flow respirometry. Bats were placed in a sealed chamber (325 ml) which was backlit from 03:30 to maintain a normal circadian rhythm, and placed inside a temperature-controlled cabinet. The closed chamber was connected to a pump (Eheim 100, EHEIM GmbH & Co., Deizisau, Germany), which supplied air from outside. Air was dried of humidity using Drierite before and after passing through the chambers, before finally entering a FOXBOX analyser (Sable Systems International, Las Vegas, NV, USA), which analyzed both *V̇*O_2_ and carbon dioxide production. The sample air also passed through an identical but empty chamber, which was used to perform baseline measurements for 15 min every hour (i.e., each set *T*_a_ consisted of 15 min of baseline measurements followed by 45 min of bat measurements). The analyser was zeroed at the onset of each field season using a 100% stock nitrogen. Additionally, the analyser was span-calibrated to 20.95% O_2_ in the middle of the first baseline at the onset of the experiment, as well as the prolonged baseline when *T*_set_ was reduced to 0 °C the following morning. Data recorded by the analyser were logged and stored in the software Expedata (Sable Systems International, Las Vegas, NV, USA) every 1 min.

To measure *T*_a_, an iButton (model DS1923-F5, Dallas Semiconductor Inc., Dallas, TX, USA) was placed in the bottom of each chamber. The iButtons recorded temperature every minute to the nearest 0.001 °C. As other models of iButtons have been shown to emit ultrasound (Willis et al. 2009), we checked for this prior to the field season using a heterodyne bat detector (Model D200, Petterson Elektronik AB, Uppsala, Sweden) and observed no indication of ultrasound noise. Additionally, iButtons were calibrated in a water bath against a precision thermometer and revealed little difference between iButtons, similar to what was reported by Davidson et al. (2003). The upper half of the chamber was covered with mesh netting, on which the bat could roost. When placed inside the chamber, the cabinet was set to 5 °C to motivate the bat to enter torpor. Bats were placed in the chamber at differing times throughout the night (mean ± SD, 160.5 ± 70.3 min after sunset). Upon arrival to the flight cage, one bat was immediately placed into the chamber and thus not handfed or supplied with water before the experiment. But digestive state presumably differed as time of capture differed between bats. At approximately 09:00, the following morning, the set temperature (*T*_set_) was reduced to 0 °C. Thereafter, the *T*_set_ was increased by 5 °C every hour until it reached 25 °C, after which we increased it by 3 °C per h until it reached 37 °C. For one measurement, technical issues in the recording computer meant we could only record data at *T*_a_ > 26 °C for one bat. The lowest mean *T*_a_ at which MR was measured was 0.99 ± 1.28 °C (± SD, *N* = 21). The highest mean *T*_a_ was 36.14 ± 1.40 °C (± SD, *N* = 22). The total length of each experiment lasted on average for 16.0 ± 1.4 h (± SD, *N* = 22).

Incurrent flow rate was set at 315 mL min^−1^ when the bat was placed in the chamber. At approximately 09:00, flow rate was reduced, as all bats were in torpor, and kept between 101 and 248 mL min^−1^ while the bat was torpid. To determine when a bat had exited torpor, we routinely observed the O_2_ measurements in the software Expedata, as well as observing via a camera in the temperature-controlled cabinet. As soon as the bat exited torpor, flow rate was increased to 315 mL min^−1^.

To calculate *V̇*O_2_, we selected a series of stable values over at least five consecutive measurements (i.e., at least 5 min) at each *T*_set_. Using the software *R* (version 4.0.2), the lowest 5 min mean within this selection was extracted using the runMean() function in the TTR package (Ulrich 2021) for further analysis. Overall, the *T*_a_ generally fluctuated slightly within each hour. At *T*_set_ of 0 °C, the *T*_a_ fluctuated above the *T*_set_, whereas between *T*_set_ of 5 and 37 °C the *T*_a_ fluctuated slightly below the *T*_set_*.* At the lowest *T*_set_, we selected at least five consecutive measurements when the *T*_a_ was lowest to calculate to which extent the bats showed active thermoregulation (Table S1).

We corrected for drift and calculated *V̇*O2 using Eq. (10.5) in Lighton (2018),

$$\dot{V}{\text{O}}_{2} = FR_{i} \left[ {\left( {F_{{\text{i}}} {\text{O}}_{2} {-}F_{{\text{e}}} {\text{O}}_{2} } \right){-}F_{{\text{e}}} {\text{O}}_{2} \left( {F_{{\text{e}}} {\text{CO}}_{2} {-}F_{{\text{i}}} {\text{CO}}_{2} } \right)} \right]/\left( {1{-}F_{{\text{e}}} {\text{O}}_{2} } \right)$$

where FR_*i*_ is the incurrent air flow rate, *F*_i_O_2_ is the fractional content of incurrent oxygen, *F*_e_O_2_ is the fractional content of excurrent oxygen, *F*_i_CO_2_ is the fractional content of incurrent carbon dioxide, while *F*_e_CO_2_ is the fractional content of excurrent carbon dioxide.

Bats were weighed (± 0.1 g) immediately before being placed in the chamber, and again immediately after the metabolic trial. As *V̇*O_2_ is very low during torpor, mass loss is correspondingly very low during torpor. Therefore, to be able to calculate a more precise value of mass-specific MR, we calculated body mass at any given time based on the equation:

$$M_{{{\text{cur}}}} = M_{{{\text{prev}}}} {-}(\dot{V}{\text{O}}_{{2{\text{cur}}}} )/(\dot{V}{\text{O}}_{{2{\text{tot}}}} )*M_{{\text{t}}}$$

where *M*_cur_ is the calculated mass at each minute, *M*_prev_ is the calculated mass in the previous minute, *V̇*O_2cur_ is the O_2_ consumption in the current minute, *V̇*O_2tot_ is the total calculated O_2_ consumption over the entire experiment, and *M*_t_ is the total loss of *M*_b_ over the entire experiment. During baseline and periods where the flow rate was too high and the oxygen analyzer therefore had problems picking up the minor *V̇*O_2_ of torpid bats, we calculated the mean *V̇*O_2_ of the 5 min before and after this period and assigned it to these periods as the *V̇*O_2_ for use in the calculations of *M*_cur_.

As we investigated the *V̇*O_2_ measurements of each individual visually, it was evident that 45% of the bats (*N* = 10) maintained torpor at higher temperatures (i.e., > 30 °C), as MR increased exponentially with increasing *T*_a_. These were considered thermoconforming within the TNZ (referred to as Group 1). The remaining bats (*N* = 12) aroused at lower *T*_a_. The latter group was defined as euthermic for the rest of their experiment as they did not reenter torpor at higher *T*_a_ (referred to as Group 2). All stable measurements at a lower *T*_a_ before arousal were considered TMR. As previous studies have shown that bats thermoregulate below 1.8–6.7 °C (Willis et al. 2005; Stawski and Geiser 2011; Currie et al. 2018), an increase in *V̇*O_2_ consumption with decreasing *T*_a_ at low ambient temperatures was defined as active thermoregulation, as they actively increased heat production.

We chose to analyse these two groups separately, as the latter group showed more stable readings over multiple temperatures within the TNZ. To quantify the arousal events, we defined an arousal as the point in time when *V̇*O_2_ in torpid bats increased substantially. The *T*_a_ measured at the same minute as metabolic rate started to increase was used as the *T*_a_ of the start of the arousal. The length of the arousal in minutes was quantified from the start of the increase to the time at which mass-specific *V̇*O_2_ peaked or stabilized. Although bats are known to overshoot their *V̇*O_2_ relative to the RMR during arousals at the same *T*_a_ (Turbill et al. 2008), the decrease phase of the arousal was not included in the analyses as it was often interrupted by baseline measurements. We used the difference in *V̇*O_2_ between the minute at which the *V̇*O_2_ peaked and the last measurement before the increase in *V̇*O_2_ started as a proxy for *V̇*O_2_ during the arousal (Figures S1 and S2).

### Statistical analyses

#### Body mass

All analyses were performed using *R* (version 4.0.2). Results are presented in the language of evidence as suggested by Muff et al. (2022).

To analyze factors influencing changes in *M*_b_ upon capture we fitted a linear model with *M*_b_ as a response variable, and days after June 1st, time after sunset (minutes), and sex as fixed effects, assuming a Gaussian error distribution. Two males were measured two times in different years, but since the recaptures were a year apart, and *M*_b_ differed highly between captures for both individuals, we treated the measurements as independent. To see if there were any differences in *M*_b_ between the bats which did not exit torpor prior to the TNZ (Group 1) and those that did (Group 2), we performed linear regression analyses with *M*_b_ at the start of the experiment as a response variable, and group as a fixed effect. Additionally, to investigate if *M*_b_ at the onset of the experiment affected the mass loss and the *M*_b_ at the end of the experiment, we fitted simple linear regressions with *M*_b_ at the end of the experiment as the response variable, and *M*_b_ at the onset of the experiment and Group as fixed effects.

#### Metabolic rate

As the timing of torpor entry differed between bats, we fitted a linear regression model using the lm() function with time spent before torpor entry in minutes as the response variable, and *M*_b_ at the onset of the experiment as the explanatory variable. To estimate how metabolic rate differs with increasing *T*_a_ in the bats which aroused at lower *T*_a_, we fitted a linear regression model using the lm() function with *V̇*O_2_ as a response variable, and *T*_a_ as an explanatory variable. Thereafter, we used Davies test, using the davies.test() function in the segmented package (Muggeo 2008) to check for the presence of a significant inflection point in the relationship between *V̇*O_2_ and *T*_a_. If an inflection point was identified, we performed a broken stick regression using the segmented() function in the segmented package (Muggeo 2008) to identify at which *T*_a_ the relationship with *V̇*O_2_ changed. To check if the segmented() function provided a better fit than the initial lm() function, we performed ANOVA analysis on both functions. After identifying a potential inflection point, we defined all measurements at *T*_a_ below the inflection point as RMR and all measurements at *T*_a_ above the inflection point as BMR.

The RMR was estimated using a linear mixed-effects model using the lmer() function (Bates et al. 2015) with *V̇*O_2_ below the inflection point as a response variable, and *T*_a_ and *M*_b_ as a fixed effects. Individual bat ID was added as a random effect. A similar analysis was performed on BMR with *V̇*O_2_ above the inflection point as a response variable. We fitted an exponential growth curve to the TMR data for each group using the nls() function in *R* and performed a linear mixed model with minimum TMR of each individual as the response variable and days after June 1st, sex, and group as fixed effects.

#### Active thermoregulation

To estimate the slope of active thermoregulation during torpor at low *T*_a_, we performed a linear mixed-effects model with *V̇*O_2_ as the response variable, and *T*_a_ at which the measurement was taken as a fixed effect. Individual ID was added as a random effect. This analysis consisted of a subset of individuals, as some individuals did not show any clear increase in *V̇*O_2_ at low *T*_a_ (*N* = 8), while in some cases the difference in *T*_a_ between two means were too big to give a reliable estimate (*N* = 3).

#### Arousal

To better understand the physiology and timing of arousals, we performed four separate analyses. First, we performed a simple *t* test to compare the *V̇*O_2_ during arousal between the two groups. Second, to estimate arousal costs with decreasing *T*_a_, we fitted a simple linear regression with *V̇*O_2_ during arousal as the response variable, and *T*_a_ as an explanatory variable. Third, we performed a simple *t* test to compare the number of minutes needed to arouse between the two groups. Fourth, we fitted a simple linear regression with the *T*_a_ at which arousal occurred as a response variable and *M*_b_ at the onset of the experiment as an explanatory variable to investigate if body condition affected arousal. This was also repeated with estimated *M*_b_ at the timing of arousal as an explanatory variable. We performed simple linear regressions as all bats in our study only aroused once per experimental run, and only completed one torpor bout.

## Results

### Body mass

**Fig. 1:**
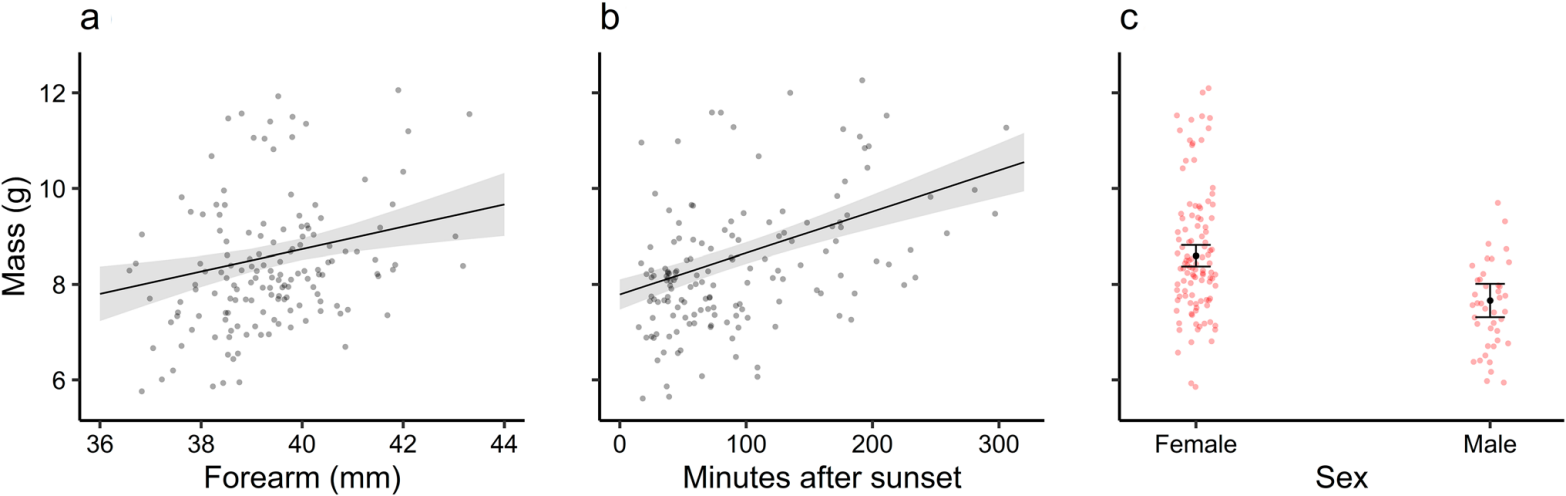
Fixed effects influencing body mass (g) in captured *P. auritus* (*N* = 89, *n* = 157) in Nittedal, Norway between 2017 and 2021. Body mass increased with **a** forearm length (mm), **b** time after sunset (min), and **c** was higher in females than in males. In **a** and **b** solid lines show estimated relationships and shaded polygons show 95% confidence limits. In **c** the black dots show the estimated average and the whiskers show the 95% CIs. Dots show measured values

Average *M*_b_ upon capture was 7.82 ± 0.96 g (range 6.6–10.7 g, *N* = 22), while forearm length averaged 39.4 ± 1.3 mm (range 36.7–43.2 mm). There was strong evidence that *M*_b_ increased with time after sunset (*M*_b_ 0.009 g min^−1^ ± SE 0.003, *p* = 0.004), but no evidence that it increased with days after June 1st (*p* = 0.86) or forearm length (*p* = 0.40), and there was no difference between sexes (*p* = 0.48). In contrast, the average *M*_b_ for all *P. auritus* (*N* = 90, *n* = 156) captured in the study area between 2017 and 2021 was 8.30 ± 1.31 g (*N* = 156), which is higher than for the bats included in the present study (*t*_33.3_ = − 2.08, *p* = 0.045). In addition to a higher *M*_b_ with time after sunset, there was also very strong evidence that *M*_b_ was positively related to forearm length, and that there was a difference between sexes (Table 1, Fig. 1). Because females have an earlier reproductive period and had greater *M*_b_ than males (females: 8.62 ± 1.32 g, *N* = 112; males: 7.49 ± 0.91 g, *N* = 44, *t*_113.4_ = 6.1, *p* < 0.0001), and as females had longer forearms (females: 39.6 ± 1.2 mm, *N* = 112; males: 38.9 ± 1.4 mm, *N* = 44, *t*_64.0_ = 3.2, *p* = 0.002) we fitted linear mixed-effects models for each sex separately (Table 1).

**Table 1:** Fitted models and model statistics for modelling body mass (g) as a function of days after June 1st, forearm length (mm); time after sunset (minutes); and sex (male or female) in brown long-eared bats (*Plecotus auritus*)

Explanatory variable	Model (group)	Estimates	SSQ	*F*	*df*	*r*^2^	*p*	*σ*^*2*^	*τ*_00_
Intercept	M1 (all)	− 1.267	68.654	0.190		0.487	0.663	0.90	0.17
Days after June 1st		− 0.003	0.834	0.930	140.6		0.337		
Forearm length		0.233	9.132	10.184	74.3		**0.002**		
Time after sunset		0.009	41.974	46.809	135.0		**< 0.001**		
Sex		− 0.933	16.713	18.639	69.1		**< 0.001**		
Intercept	M2 (Females)	− 2.646	40.564	0.433		0.437	0.514	1.01	0.29
Days after June 1st		− 0.005	1.774	1.753	104.0		0.188		
Forearm length		0.271	7.257	7.170	43.3		**0.010**		
Time after sunset		0.009	31.533	31.153	85.9		**< 0.0001**		
Intercept	M3 (Males)	− 0.233	10.107	− 0.005		0.469	0.946	0.46	0.06
Days after June 1st		0.001	0.072	0.157	31.4		0.694		
Forearm length		0.178	1.875	4.117	20.4		0.056		
Time after sunset		0.008	8.159	17.913	33.3		**0.0002**		

P values of explanatory variables which had a strong effect on body mass are presented in bold

Results from linear mixed models with continuous response assuming Gaussian error distribution. M1 show fitted model for both sexes combined. M2 and M3 show fitted models and associated model statistic for females and males, respectively

**Fig. 2:**
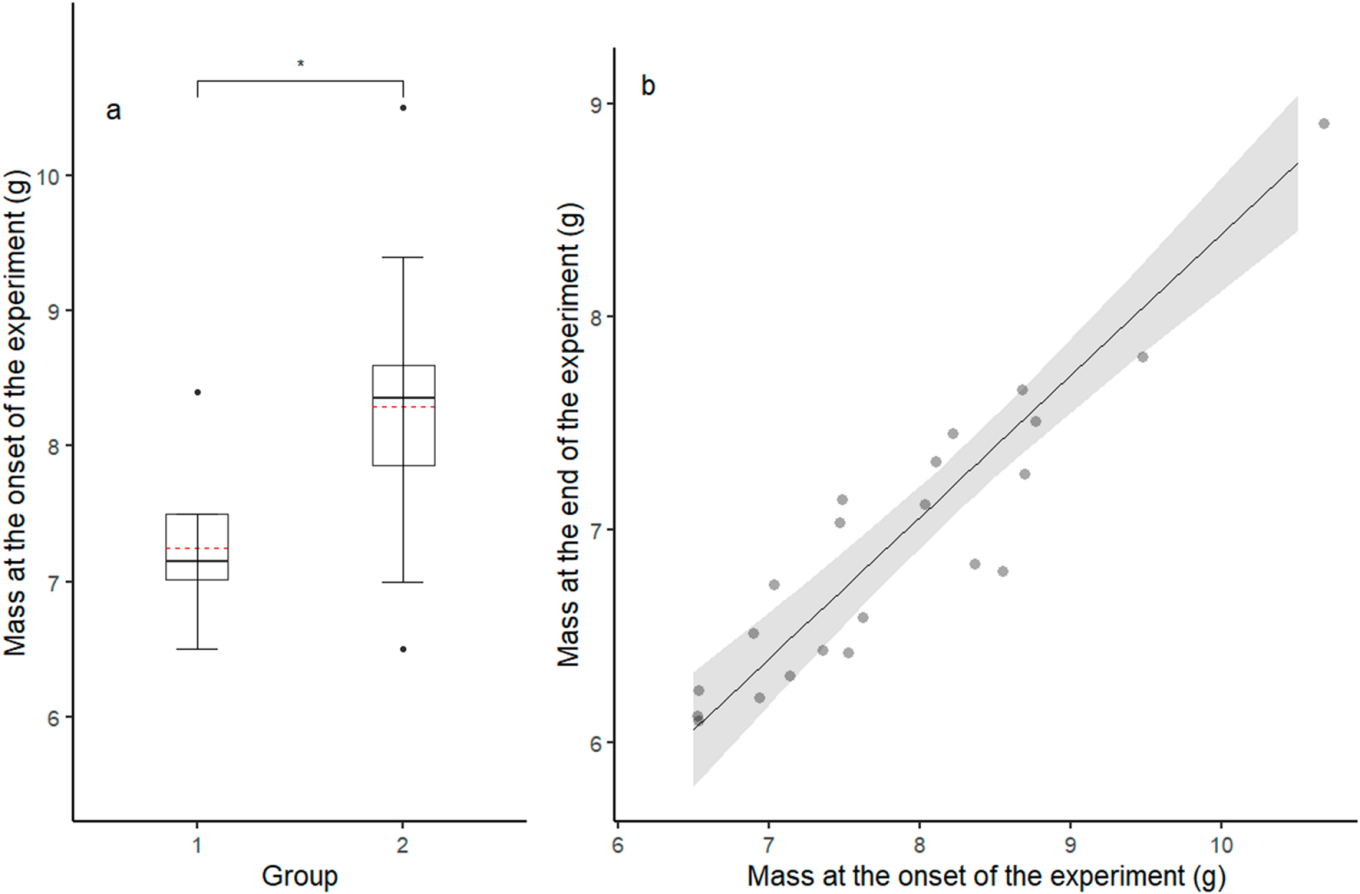
Body mass (g) at **a** the onset of the experiment was higher in bats that exited torpor before the estimated lower critical temperature (*T*_lc_) of the TNZ (Group 2), than those that did not (Group 1). Body mass (g) at **b** the end of the experiment was higher in bats with a higher body mass at the onset of the experiment (*g* = 1.73 + 0.67 × *g*, *r*^2^ = 0.87*, p* < 0.005)

At the onset of the experiment, there was strong evidence that average *M*_b_ for the bats that aroused below *T*_lc_ (8.29 ± 1.04 g, *N* = 12, Group 2) was higher (*p* = 0.009) than for the bats that aroused above *T*_lc_ (7.25 ± 0.53 g, *N* = 10, Group 1, Fig. 2a). There was strong evidence that *M*_b_ at the end of the experiment increased with higher *M*_b_ at the onset of the experiment (*g* = 1.73 + 0.67 × *g*, *r*^2^ = 0.87*, p* < 0.005, Fig. 2b), but did not differ between the bats which aroused from torpor prior to entering the TNZ and those that did not (*p* = 0.88). *M*_b_ showed a noticeable decrease at the onset of the experiment, followed by a long period in which the *M*_b_ remained stable during torpor, until it decreased gradually at higher *T*_a_ (Figures S1 and S2).

## Metabolic rate

**Fig. 3:**
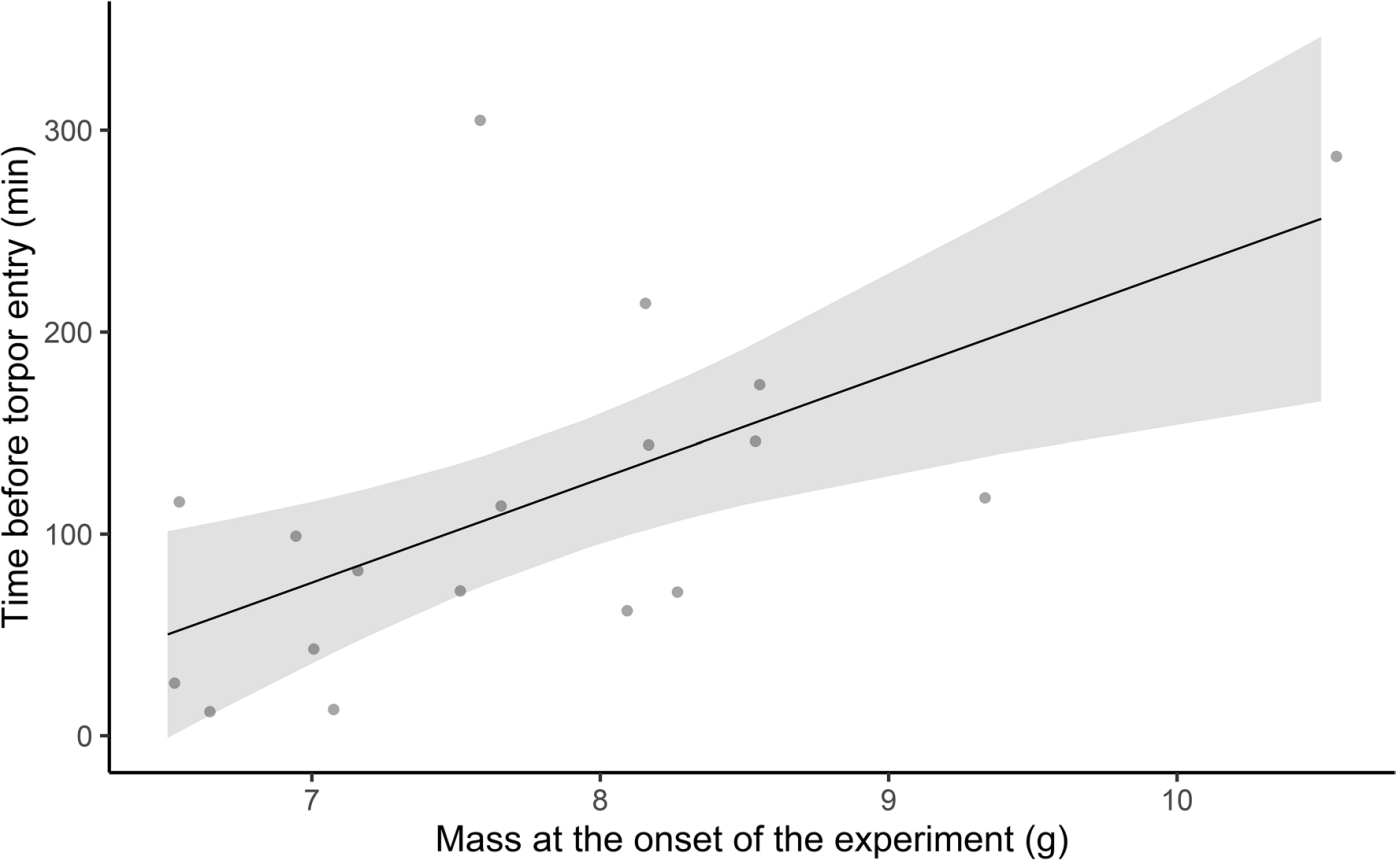
Bats with a higher body mass (g) spent more time exploring the new environment before entering torpor. Solid lines show estimated relationships, shaded polygons show 95% confidence limits and dots show measured values

**Fig. 4:**
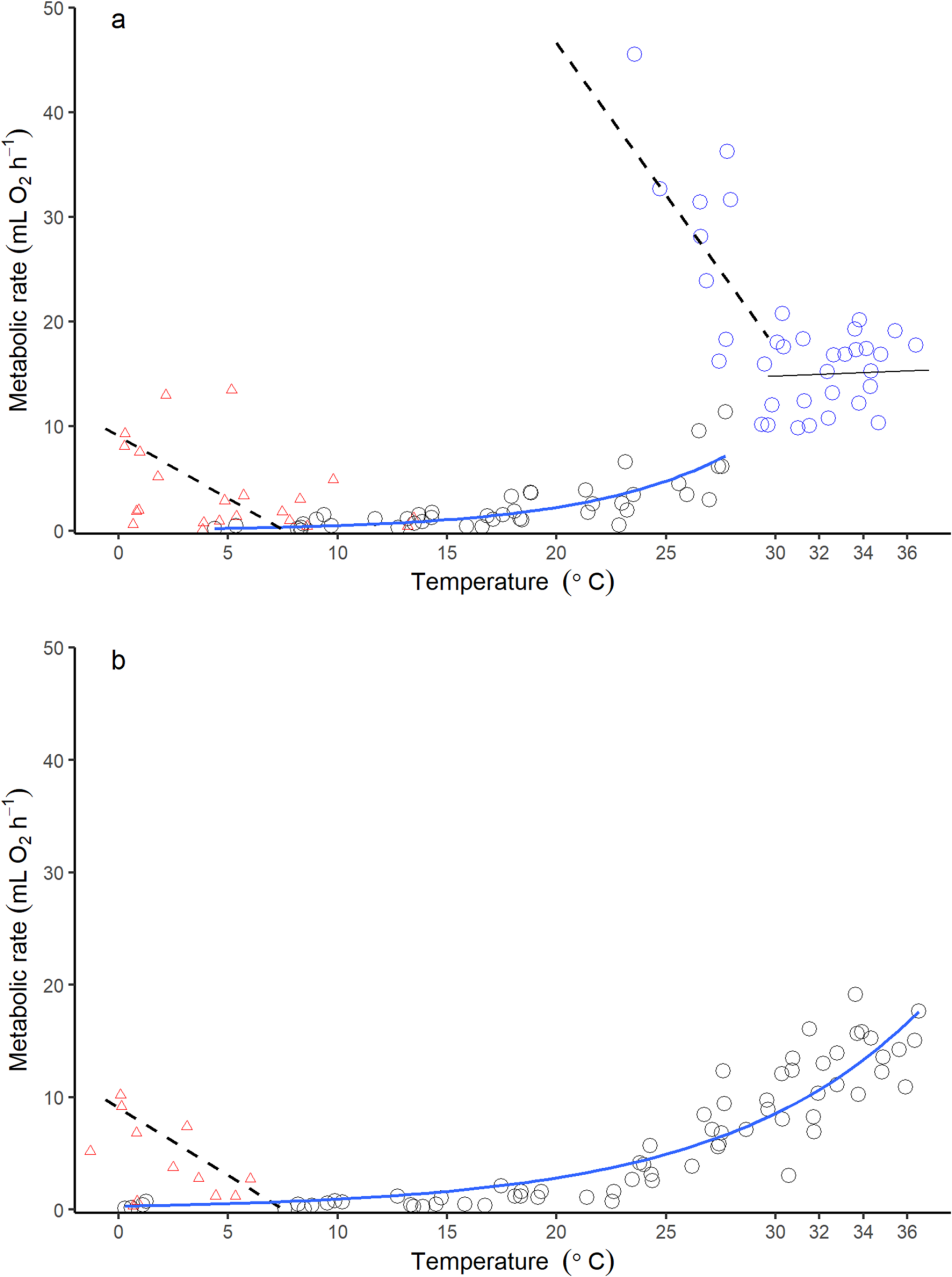
Metabolic rate (mL O_2_ h^−1^) as a function of ambient temperature (*T*_a_ °C). Metabolic rate of bats which **a** exited torpor before the TNZ, and bats which **b** remained torpid at temperatures above *T*_lc_. The dotted line in **a** shows the increase in metabolism with decreasing *T*_a_ (2.91 mL O_2_ h^−1^ °C^−1^, *N* = 8, *n* = 12) for euthermic bats (RMR). While the dotted line present in both **a** and **b** at *T*_a_ below 6.73 °C shows the increase in metabolism with decreasing *T*_a_ (1.30 mL O_2_ h^−1^ °C^−1^, *N* = 10, *n* = 21). Blue circles show measurements of included in broken stick regression for RMR and BMR (15.5 ± 3.4 mL O_2_ h^−1^, *N* = 11, *n* = 24), indicated by the black line which also shows the range of the TNZ (29.67–36.4 °C). Black circles in both plots show measurements of TMR. Blue line in both plots indicate the exponential growth curve of TMR with increasing *T*_a_ (**a**), 0.127 × 1.148^Ta^ and **b** 0.141 × 1.151^Ta^. Red triangles show plots where bats showed active thermoregulation (colour figure online)

Bats entered torpor after 102.8 ± 85.3 min (± SD, *N* = 19) into the experiment. Torpor entry occurred later in bats that weighed more at the onset of the experiment (minutes = − 284.2 + 51.5 × *g*, *r*^2^ = 0.36, *p* = 0.005, *N* = 19, Fig. 3). Broken stick regression revealed that *V̇*O_2_ decreased until 29.7 °C and provided a better fit than the linear model (*F* = 42.5 vs *F* = 23.2, respectively). RMR increased with decreasing temperature (mL O_2_ h^−1^: 119.1 − 2.94 × *T*_a_, *p* = 0.002, *N* = 8, *n* = 12), but was not affected by *M*_b_ (*p* = 0.658). We, therefore, removed *M*_b_ from the model and repeated the analysis (mL O_2_ h^−1^: 104.9 − 2.9 × *T*_a_, *p* = 0.002, *N* = 8, *n* = 12, Fig. 4a). BMR was affected by neither *M*_b_ (*p* = 0.403), nor *T*_a_ (*p* = 0.549), and was estimated at 15.5 ± 3.4 mL O_2_ h^−1^ (*N* = 11, *n* = 24). TNZ was between 29.7 and at least up to 36.4 °C. Four individual bats measured had an increased MR at 36.4 ± 0.6 °C (range 35.7–37.0 °C).

During torpor, *V̇*O_2_ increased exponentially with increasing temperatures for bats that exited torpor before the TNZ was reached (mL O_2_ h^−1^ = 0.127 × 1.148^Ta^, *r*^2^ = 0.65, *N* = 12, *n* = 43, Fig. 4a). A similar relationship was found for bats that remained torpid within the TNZ (mL O_2_ h^−1^ = 0.141 × 1.151^Ta^, *r*^2^ = 0.86, *N* = 10, *n* = 86, Fig. 4b). The minimum measured TMR (0.644 ± 0.493 O_2_ h^−1^, *N* = 21) occurred at 10.1 ± 5.2 °C (*N* = 21) but varied between individuals (range 0.3–18.4 °C). The minimum measured TMR per individual was not related to days after June 1st, nor did it differ between bats that aroused before the *T*_lc_ and those that did not, or between males and females (*p* = 0.756).

## Active thermoregulation during torpor

When exposed to *T*_a_s below 6.7 °C, as indicated by the intercept of the thermoregulatory curve of thermoregulating bats and the exponential curve of TMR, bats which showed active thermoregulation increased TMR by 1.30 mL O_2_ h^−1^ °C^−1^ (*N* = 10, *n* = 21; Fig. 4). Average *M*_b_ of the bats that increased *V̇*O_2_ at low *T*_a_ was marginally higher (8.34 ± 1.06 g, *N* = 10) at the onset of the experiment then the average *M*_b_ of those that did not increase *V̇*O_2_ (7.46 ± 0.81, *N* = 8, *t*_*16.0*_ = 1.99, *p* = 0.064).

## Arousal

**Fig. 5:**
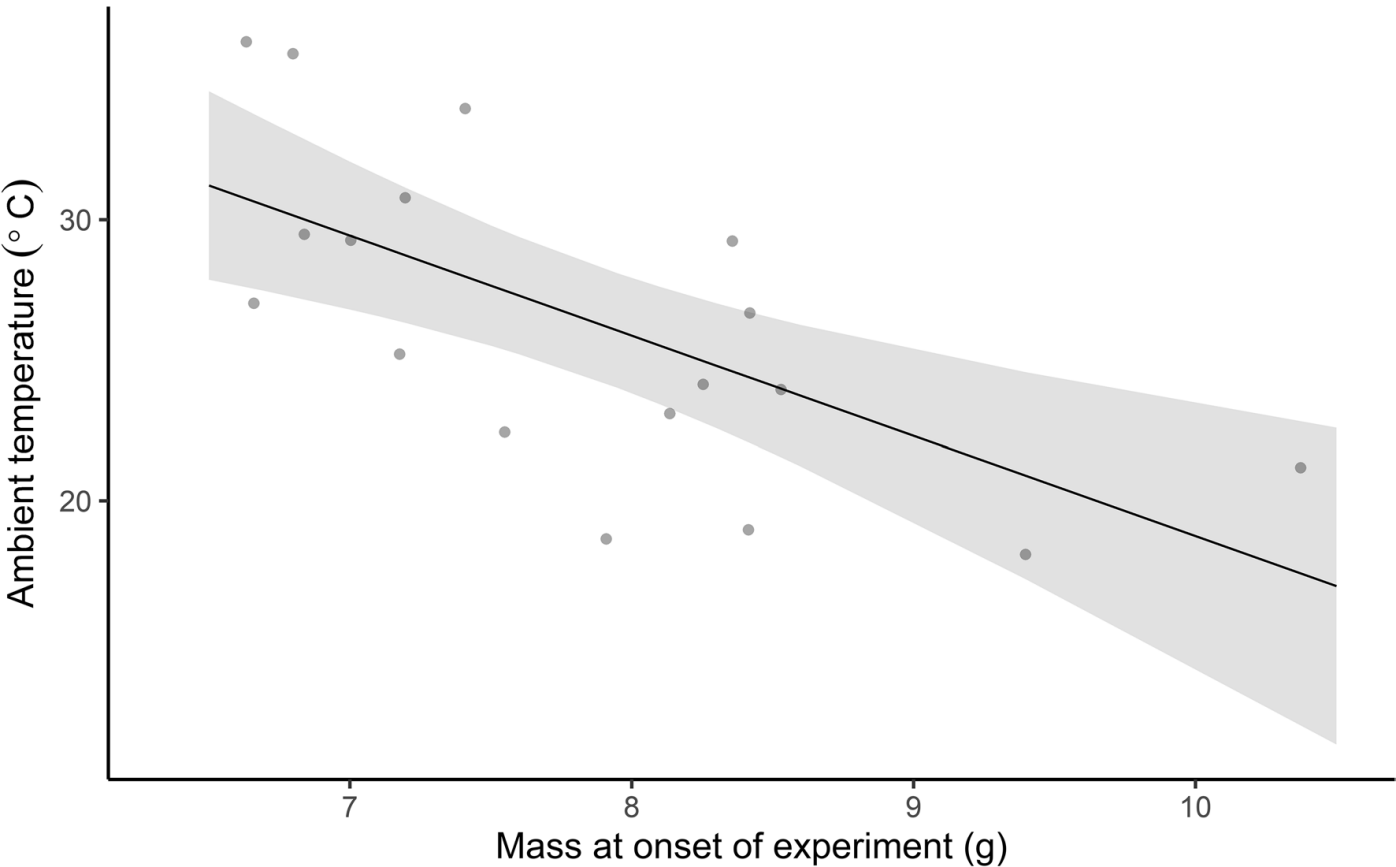
The ambient temperature (*T*_a_ °C) at which bats aroused decreased (*y* = 58.08 − 4.42*x*, *p* = 0.004, *N* = 18) with increasing body mass (g) at the onset of the experiment

Bats that exited torpor below the *T*_lc_ aroused at 22.9 ± 3.5 °C (*N* = 11), while bats that remained torpid into the TNZ aroused at 31.9 ± 3.7 °C (*N* = 7). In four cases, arousal was either not apparent, or occurred during baseline measurements. There was strong evidence for a higher *V̇*O_2_ during arousal (*t*_*11.24*_ = 3.96, *p* = 0.002) in the bats that aroused at a lower temperature (73.78 ± 40.99 mL O_2_ h^−1^), compared to those that aroused later (23.44 ± 7.97 mL O_2_ h^−1^)_*,*_ and increased with decreasing *T*_a_ (mL O_2_ h^−1^ = 198.38 − 5.46 × *T*_a_, *p* < 0.001). The number of minutes the bats needed to arouse did not differ between the bats which aroused at *T*_a_ < 29.7 °C, and those that aroused at *T*_a_ > 29.7 °C (*t*_11.09_ = − 1.39, *p* = 0.19). Bats with higher *M*_b_ at the onset of the experiment aroused at lower ambient temperatures (*T*_a_ = 54.38 − 3.56 × g, *p* = 0.003, Fig. 5b), but also had a higher estimated *M*_b_ when arousal occurred (*T*_a_ = 58.08 − 4.42 × g, *p* = 0.004), despite having delayed torpor entry for longer at the onset of the experiment.

## Discussion

By studying how *P. auritus* close to the northern range of its distribution respond physiologically to different temperature conditions, we found that individual state influenced quantitative energetics in this species. As predicted, individual bats with a higher *M*_b_ aroused from torpor at lower *T*_a_, while individuals with a lower *M*_b_ postponed arousal to higher *T*_a_. Although we observed substantial active thermoregulation at low temperatures in almost half of the measured bats, the relationship between increased *V̇*O_2_ at low *T*_a_ and *M*_b_ was unclear, with only a marginal difference in *M*_b_.

*M*_b_ at time of capture increased as expected with time after sunset. As bats at high latitudes are subject to short nights for foraging during summer (Frafjord 2013; Michaelsen et al. 2018), they need to build up a large energy reserve on an almost daily basis to survive and reproduce. At the same time, they stay in the roost for approximately 20 h each day. Thus, depending on the amount of time during the day spent torpid, they generally leave the roost with a relatively low *M*_b_ as they will have consumed a large portion of their gut fill.

TMR was similar between bats that aroused before the *T*_lc_, and those that did not. TMR increased exponentially with increasing *T*_a_, and is similar to that of equally sized vespertilionid bats in the southern hemisphere (Geiser and Brigham 2000; Turbill et al. 2008; Stawski and Geiser 2011). Thus, further supporting the notion that torpor in subtropical vespertilionid bats does not differ from temperate vespertilionid bats (Stawski and Geiser 2011; Fjelldal et al. 2022), as torpor is a mechanism to cope with energy limitation at low *T*_a_ regardless of the habitat.

Interestingly, only half of the bats in the present study showed signs of active thermoregulation during torpor at low *T*_a_, despite all 21 bats being exposed to *T*_a_ below the estimated critical *T*_a_ of 6.7 °C. However, bats which showed active thermoregulation only had a marginally higher *M*_b_ at the onset of the experiment compared to those that did not. In a study conducted by Currie et al. (2018) where both RMR and the torpor thermoregulation curve of bats was estimated, the metabolic rate of both curves had an equal increase. A trend which is similar to what is found in other animals that employ torpor (see Geiser 2021). In contrast, in our study, active thermoregulation at low *T*_a_ was with a much shallower increase in TMR than what was observed in resting bats (i.e., RMR).

In the literature, the critical *T*_a_ is often referred to as a species or population specific temperature threshold, which bats have evolved as long-term adaptations to their respective environmental conditions (Stawski and Geiser 2011). However, as the critical *T*_a_ estimated in the present study is higher than the *T*_a_ in which *P. auritus* regularly hibernate at this latitude (Wermundsen and Siivonen 2010), and considerably higher than the lowest measured *T*_b_ of − 2 °C (Eisentraut 1956), this indicates that individual bats may choose the level of thermoregulation to some extent at different *T*_a_ to avoid torpor-related costs. A lower increase in *V̇*O_2_ when thermoregulating at low *T*_a_ may be related to a higher level of thermal insulation. As the length of fur coat is equal during the individual experiments here, the assumed difference in insulation could be due to a greater peripheral part of the body being kept cold, and *T*_b_ is only defended in vital parts of the body. Due to this increase in insulation, bats will only need to increase *V̇*O_2_ to a lesser extent to prevent tissue damage to vital organs. It is also worth noting that our measurements were performed during summer, as bats may defend a higher *T*_b_ at this time of year as opposed to during the hibernation season. But the question as to why some bats choose to thermoregulate and some do not, remains unanswered.

It is becoming more and more clear that *M*_b_ not only affects metabolism but can also affect the metabolic strategy of the animal. As observed here, bats with higher *M*_b_ readily spent more energy to delay torpor. All bats in our study showed similar TMR when thermoconforming but differed in level of exploration at the onset of the experiment, active thermoregulation at lower *T*_a_, and timing of arousal. Bats with a higher *M*_b_ at the onset of the experiment aroused at lower *T*_a_, and lost more *M*_b_ during the experiment. This loss in *M*_b_ was also related to these bats delaying torpor for a longer period at the onset of the experiment. Similarly, recently fed *Myotis lucifugus* delay torpor entry independent of temperature (Matheson et al. 2010). Similar behavioural responses have been seen in edible dormice (*Glis glis*), where heavier animals aroused more frequently and stayed euthermic for longer (Bieber et al. 2014), and woodchucks (*Marmota monax*), which defended a higher *T*_b_ when more energy was available (Zervanos et al. 2013).

The delay of torpor in recently fed bats, along with an earlier arousal, is presumably related to a trade-off between different costs and benefits of torpor. Although there are potential costs of maintaining torpor for longer time periods, such as sleep deprivation (Humphries et al. 2003), predation (Estok et al. 2010; Haarsma and Kaal 2016), and buildup of waste materials (Thomas and Geiser 1997; Ben-Hamo et al. 2013; Landes et al. 2020), the physiological benefits include water and energy conservation. Thus, on a day-to-day basis during the active season of the year, the physiological benefits of limiting energy consumption and water loss is likely to outweigh the costs.

As bats in our study area, and at similar latitudes, will daily spend up to 20 h in roosts without access to water (i.e., barns or trees), the risk of dehydration is potentially a contributing reason to remain in torpor during daytime. As water consumption prior to the experiment was not controlled for, *M*_b_ could also be affected by hydration level in individual bats. This effect may be exacerbated in typical studies of metabolic rate, as dry air will lead to an increased evaporative water loss.

Another potential explanation for the earlier arousal can be that heavier bats exert the reserve energy to allow for restorative activities, such as sleep (Humphries et al. 2003), protein synthesis (Heldmaier et al. 2004; Landes et al. 2020), or even digestion of previously consumed food (Turbill et al. 2008). As all bats left fecal droppings in the chamber during the experiment, some digestion must have occurred. It is, however, unknown whether this occurred prior to torpor entry, following arousal, or both. Thus, reinitiating digestion and being able to move away from potential threats in an unfamiliar environment may explain why bats with larger *M*_b_ opted for leaving torpor at an earlier stage. Additionally, in a wider context, it may be beneficial for individual bats to arouse earlier to allow for social interactions, such as grooming (Chaverri et al. 2018) and information transfer (Gager 2018). Thus, the optimal timing of arousal from torpor is probably a trade-off between the physiological benefits of torpor and the ecological costs of missing out on the benefits of euthermia. Accordingly, the condition of an individual bat should affect its decision-making under these opposing pressures.

In summary, the present study highlights how metabolism of individual bats is affected by their condition, as bats with a larger energy reserve readily arouse at lower *T*_a_, possibly to counteract the negative effects of torpor. Similarly, active thermoregulation occurred in only half of all measured bats and at *T*_a_s higher than what they normally experience during hibernation. Additionally, the increase in metabolic rate during active thermoregulation when torpid has a shallower slope compared to that of normothermic bats resting at similar temperatures below the TNZ. This indicates that the bats altered their thermal conductance by an increased insulation. Essentially, when and to which extent individual bats actively increase heat production may be behaviorally flexible, and not physiologically fixed.

## Supplementary Information

Below is the link to the electronic supplementary material.

Supplementary file1 (DOCX 89 KB)

## Data Availability

The data collected and analysed during the current study are available from the corresponding author on reasonable request.

## Abbreviations

*T*_b_: Body temperature
*T*_a_: Ambient temperature
TMR: Torpid metabolic rate
TNZ: Thermoneutral zone
*M*_b_: Body mass
*V̇*O_2_: Oxygen consumption
*T*_set_: Set temperature
BMR: Basal metabolic rate
RMR: Resting metabolic rate
*T*_*lc*_: Lower critical temperature
