# Supplementary material for: State dependence of arousal from torpor in brown long-eared bats (Plecotus auritus)
Source: J Comp Physiol B. 2022 Aug 16;192(6):815–27. doi: 10.1007/s00360-022-01451-8 (PMC9550697; doi:10.1007/s00360-022-01451-8)
Supplement: Supplementary file 1 — Supplementary file1 (DOCX 89 KB) [file 360_2022_1451_MOESM1_ESM.docx]

**State dependence of arousal from torpor in Brown long-eared bats (*Plecotus auritus*)**

Rune Sørås^1,^*, Mari Aas Fjelldal^1^, Claus Bech^1^, Jeroen van der Kooij^2^, Karoline H. Skåra^1,3^, Katrine Eldegard^4^, Clare Stawski^1,5^

^1^Department of Biology, Norwegian University of Science and Technology, Trondheim, NO-7491, Norge.

^2^Nature eduction, research and consultancy van der Kooij, Rudsteinveien 67, Slattum, NO-1480, Norway.

^3^Centre for Fertility and Health, Norwegian Institute of Public Health, P.O. Box 222 Skøyen, NO-0213 Oslo, Norway

^4^Faculty of Environmental Sciences and Natural Resource Management, Norwegian University of Life Sciences, Box 5003, Ås, NO-1433 Norway.

^5^School of Science, Technology and Engineering, University of the Sunshine Coast, Maroochydore DC, Queensland, 4558, Australia

*Author of correspondence ([rune.soras@ntnu.no](mailto:rune.soras@ntnu.no))

# Supplementary information

**Figure S1** Example of one male *Plecotus auritus* which exited torpor prior the TNZ. Top panel shows mass-specific metabolic rate (*V̇O_2_*, mL O_2_ h^-1^ g^-1^), with each color indicating each *T_set_*. Start and peak of arousal is indicated by red arrows. *V̇O_2_* from when the bat was placed in the chamber and the experiment started has been excluded. The middle panel shows estimated mass loss over the course of the experiment. The lower panel shows the variation in *T_a_* over the course of the experiment, with each *T_set_* illustrated with the same colors as in the upper panel.

**Figure S2** Example of one male *Plecotus auritus* that exited torpor after entering the TNZ. Top panel shows mass-specific metabolic rate (*V̇O_2_*, mL O_2_ h^-1^ g^-1^), with each color indicating each *T_set_*. Start and peak of arousal are indicated by red arrows. *V̇O_2_* from when the bat was placed in the chamber and the experiment started has been excluded. The middle panel shows estimated mass loss over the course of the experiment. The lower panel shows the variation in *T_a_* over the course of the experiment, with each *T_set_* illustrated with the same colors as in the upper panel.


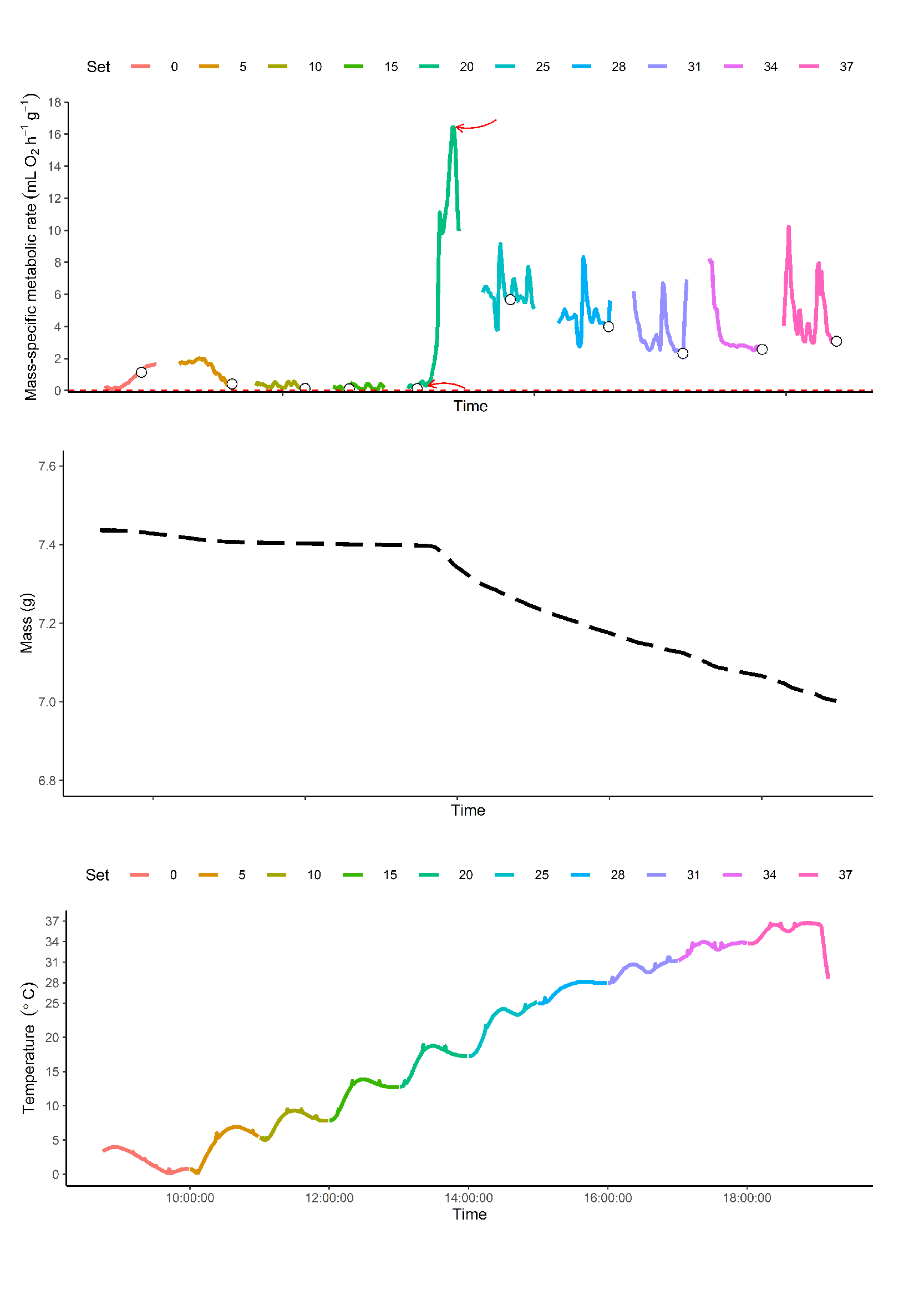


Figure S1


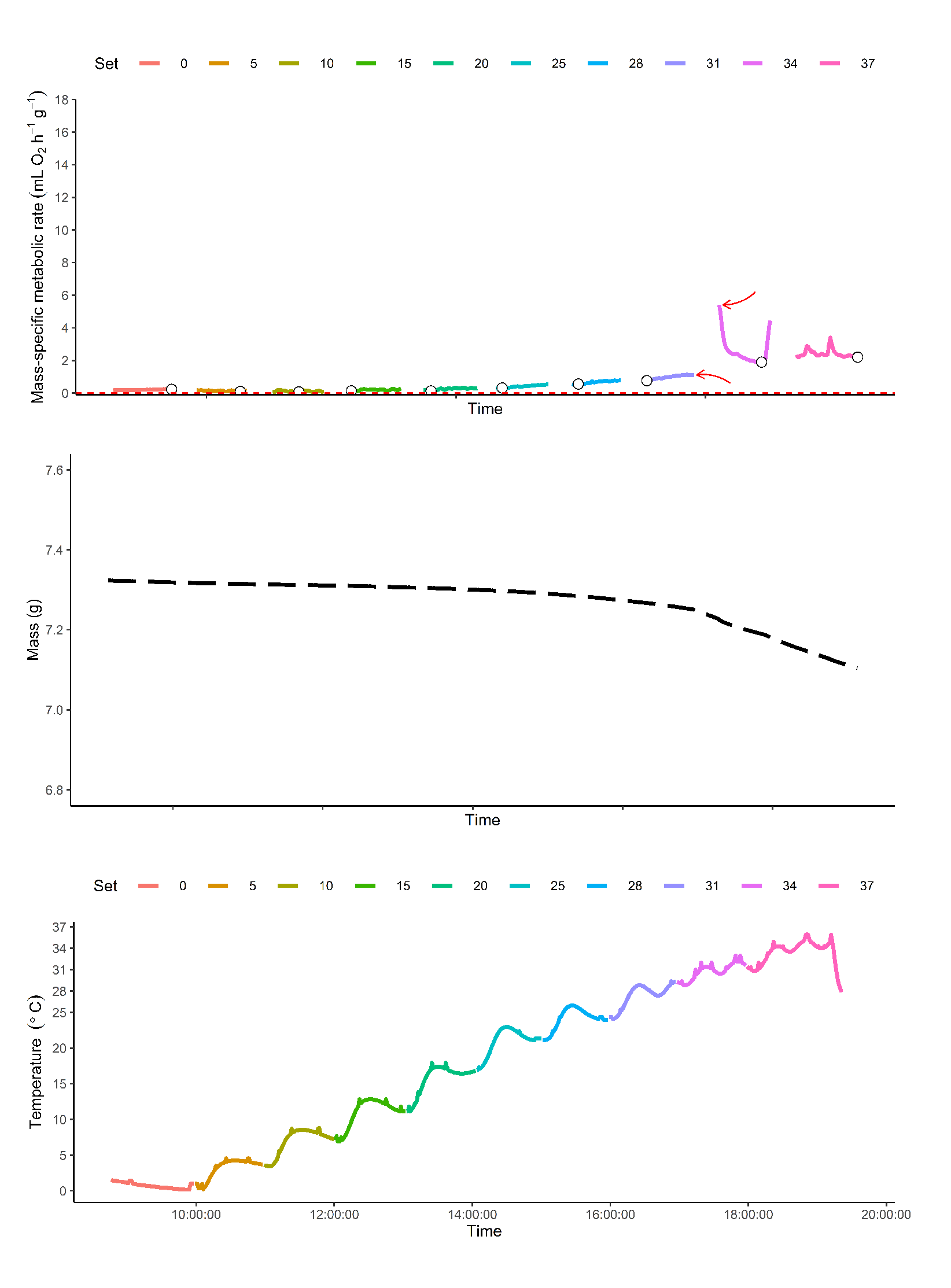


Figure S2

Table S1 Summary information of mean ± SD torpor metabolic rate (TMR), thermoregulation of torpid bats (TMR_th_), resting metabolic rate (RMR), basal metabolic rate (BMR), and ambient temperature (*T_a_*) at each Set temperature (*T_set_*). Number of samples (*N*) at each *T_set_* is also included.

| *T_set_* | 0°C | 5°C | 10°C | 15°C | 20°C | 25°C | 28°C | 31°C | 34°C | 37°C |
| --- | --- | --- | --- | --- | --- | --- | --- | --- | --- | --- |
| TMR | 0.36 ± 0.42 | 0.17 ± 0.11 | 0.11 ± 0.13 | 0.11 ± 0.05 | 0.18 ± 0.11 | 0.44 ± 0.37 | 0.78 ± 0.36 | 1.20 ± 0.44 |  |  |
| *T_a_* | 0.85 ± 1.47 | 4.15 ± 1.89 | 8.75 ± 0.69 | 13.6 ± 0.76 | 17.8 ± 1.0 | 23.0 ± 1.0 | 26.7 ± 0.97 | 29.5 ± 1.43 |  |  |
| *N* | 6 | 15 | 18 | 16 | 19 | 17 | 13 | 10 |  |  |
| TMR_th_ | 0.89 ± 0.39 | 0.86 ± 0.63 |  |  |  |  |  |  |  |  |
| *T_a_* | 1.09 ± 0.87 | 5.44 ± 0.39 |  |  |  |  |  |  |  |  |
| *N* | 9 | 2 |  |  |  |  |  |  |  |  |
| RMR |  |  |  |  |  | 4.84 ± 1.18 | 3.17 ± 0.90 |  |  |  |
| *T_a_* |  |  |  |  |  | 24.2 ± 0.82 | 27.3 ± 0.60 |  |  |  |
| *N* |  |  |  |  |  | 2 | 7 |  |  |  |
| BMR |  |  |  |  |  |  |  | 1.61 ± 0.60 | 1.75 ± 0.46 | 1.90 ± 0.24 |
| *T_a_* |  |  |  |  |  |  |  | 29.7 ± 1.27 | 32.7 ± 0.95 | 35.1 ± 0.94 |
| *N* |  |  |  |  |  |  |  | 11 | 21 | 15 |
